# A unified dinucleotide alphabet describing both RNA and DNA structures

**DOI:** 10.1093/nar/gkaa383

**Published:** 2020-05-14

**Authors:** Jiří Černý, Paulína Božíková, Jakub Svoboda, Bohdan Schneider

**Affiliations:** Institute of Biotechnology of the Czech Academy of Sciences, BIOCEV, CZ-252 50 Vestec, Prague-West, Czech Republic

## Abstract

By analyzing almost 120 000 dinucleotides in over 2000 nonredundant nucleic acid crystal structures, we define 96+1 diNucleotide Conformers, NtCs, which describe the geometry of RNA and DNA dinucleotides. NtC classes are grouped into 15 codes of the structural alphabet CANA (Conformational Alphabet of Nucleic Acids) to simplify symbolic annotation of the prominent structural features of NAs and their intuitive graphical display. The search for nontrivial patterns of NtCs resulted in the identification of several types of RNA loops, some of them observed for the first time. Over 30% of the nearly six million dinucleotides in the PDB cannot be assigned to any NtC class but we demonstrate that up to a half of them can be re-refined with the help of proper refinement targets. A statistical analysis of the preferences of NtCs and CANA codes for the 16 dinucleotide sequences showed that neither the NtC class AA00, which forms the scaffold of RNA structures, nor BB00, the DNA most populated class, are sequence neutral but their distributions are significantly biased. The reported automated assignment of the NtC classes and CANA codes available at dnatco.org provides a powerful tool for unbiased analysis of nucleic acid structures by structural and molecular biologists.

## INTRODUCTION

**Figure 1.**
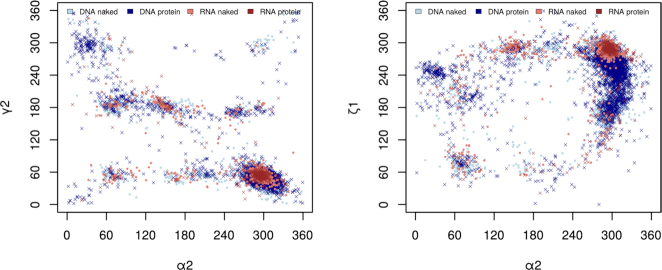
Examples of two-dimensional scattergrams of three backbone torsion angles in RNA and DNA molecules. Shown are the values from crystal structures with resolution better than 1.8 Å. The scattergram on the left plots distributions of the torsions at the backbone bonds P-O5′ (axis α2) and C5′–C4′ (axis ɣ2), the scattergram on the right distributions of the torsions at the bonds P-O5′ (axis α2) and O3′-P (axis ζ1).

Large folded RNA molecules and plastic but more uniform DNA duplexes are strikingly different and this structural diversity reflects variability of their biological functions. The overall architecture of DNA is characterized by its helicity locked in by the dominant canonical Watson–Crick base pairing and van der Waals stacking while topologically more complicated features such as hairpins, bulges or quadruplexes form more or less local disruptions. In contrast, RNA molecules display complex 3D architectures with an abundance of non-canonical base pairing motifs. These noticeable differences between the molecular architecture of RNA and DNA molecules are however much less obvious when we analyze structural behavior of these molecules at a local level, for instance as distributions of the backbone torsion angles. The scattergrams of the torsion distributions at the phosphodiester bonds O3′-P and P-O5′, and at bonds P-O5′and O5′-C5′ (Figure 1) show overlaps of the populated regions suggesting that a development of a consolidated protocol describing the structural behavior of both molecules, at least at the local level, may be possible.

The local per-residue conformational diversity of proteins is based on the well-known Ramachandran plot (1) and the following concepts of helices, sheets, loops, and turns have been routinely used for half a century. These elements of protein secondary structure have been incorporated into several protein *structural alphabets* since the late eighties (2,3) with the alphabet extent varying from eight letters of the DSSP system (4) to 13 symbols of the SST (5), and to 16 symbols of the Peptide Blocks (PB) alphabet (6). The complexities of the nucleic acid local geometry had been understood early on (7) but for a long time afterwards the topic attracted much less attention. The situation changed when large RNA ribozyme and ribosome structures started to emerge in the late nineties and several independent initiatives started to analyze the structural variability of nucleic acid fragments beyond the traditional but inadequate A/B/Z architectural classes (8–11). The fragments used in these analyses compromised between the size and available structural data of acceptable quality and the currently accepted standard for a geometry classification of nucleic acids converged to a single-stranded dinucleotide-like fragment, ‘suite’ in RNA description (8,12) or a similarly sized but geometrically more detailed fragment describing also the base orientation relative to the ribose or deoxyribose sugar ring (13,14). A different approach to the fragment definition is based on the reduced representation of the backbone by pseudo-torsions (10). This method is successful in the search for some structural motifs but it cannot discriminate important features of intermolecular interactions depending on the atomic details such as charge distribution, hydrophobic patches, or hydration patterns, and is of limited use for model building and refinement of nucleic acids. However, all these projects suffered from the one-sighted view by analyzing just one of the two nucleic acid species, either DNA or RNA.

RNA structural description has been largely motivated by identification and classification of structural motifs. Some of them, such as the kink-turn motif (15), were discovered by empirical analysis of refined structures but most by systematic bioinformatic analysis of base pairing patterns (16) or other base-related structural features (17–19). A wealth of web services and databases describing base-related RNA features is available, among others the RNA 3D Motif Atlas (20), RNApdbee (21), Rna3Dmotif (22), RNAMotifScan (23,24) and RNA Bricks (25). We believe that these and other approaches based on base or base-pairing patterns and their topological analysis need to be complemented by methods studying the geometry of local segments of nucleic acid single strands and that both approaches will ultimately merge.

**Figure 2.**
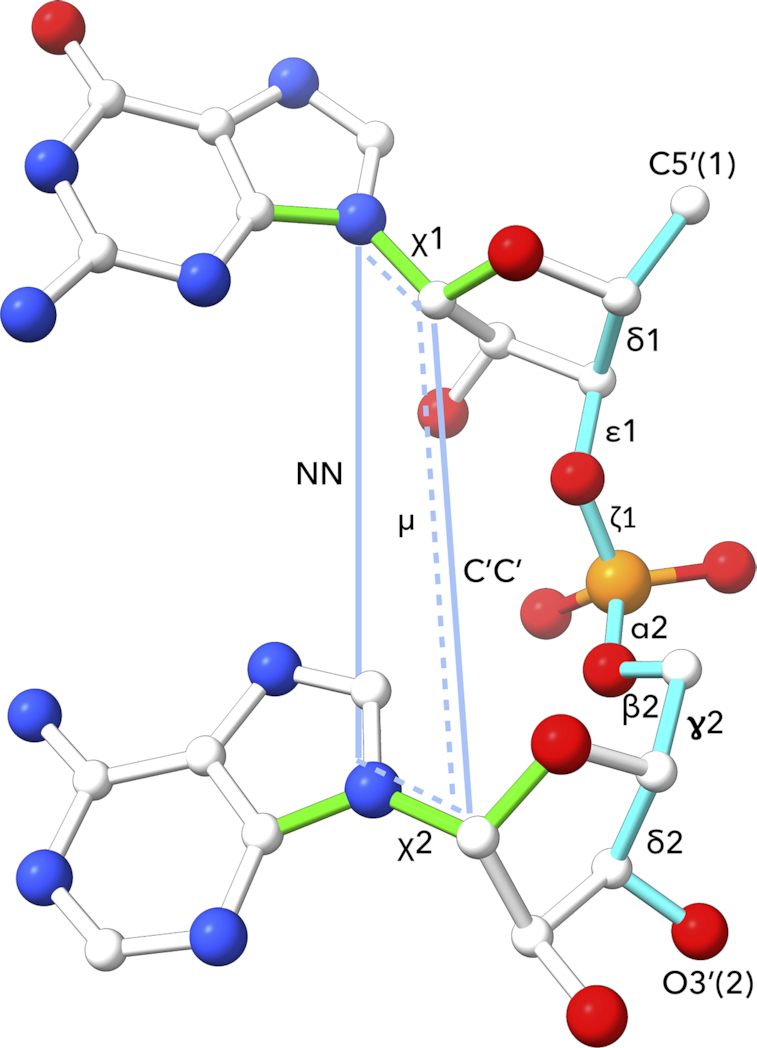
The analyzed fragment is defined by twelve geometric parameters: seven backbone torsion angles δ1 to δ2, which are highlighted in cyan, plus two torsions around the glycosidic bonds χ1and χ2 (highlighted in green), plus three parameters highlighted in light blue, one pseudo-torsion angle μ, and two distances NN and C’C’. The parameters are defined as follows: δ1 C5′(1)–C4′(1)–C3′(1)–O3′(1), ϵ1 C4′(1)–C3′(1)–O3′(1)–P(2), ζ1 C3′(1)–O3′(1)–P(2)–O5′(2), α2 O3′(1)–P(2)–O5′(2)–C5′(2), β2 P(2)–O5′(2)–C5′(2)–C4′(2), ɣ2 O5′(2)–C5′(2)–C4′(2)–C3′(2), δ2 C5′(2)–C4′(2)–C3′(2)–O3′(2), χ1 O4′(1)–C1′(1)–N1/9(1)–C2/4(1), χ2 O4′(2)–C1′(2) N1/9(2)–C2/4(2), the parameters NN as N1/9(1)–N1/9(2), C′C′ as C1′(1)–C1′(2) distances. Finally, the pseudo-torsion μ is defined as the torsion between atoms defining the glycosidic bonds of the first and second nucleotide N1/N9(1)–C1′(1)–C1′(2)–N1/N9(2).

In this work, we present an approach enabling unified analysis of both RNA and DNA structures. It is based on a classification of dinucleotide fragments (Figure 2) into one of the 97 diNucleotide Conformational classes, so called NtC classes. The classification protocol clusters structures of the dinucleotide sugar-phosphate backbone linked to the nitrogenous bases by the automated, strictly geometric protocol. Based on the new set of NtCs capable of describing both RNA+DNA structures, we generalized the structural alphabet of nucleic acids CANA (Conformational Alphabet of Nucleic Acids, (14)) so that the newly defined CANA codes better reflect the structural diversity of nucleic acid molecules beyond the outdated and for RNA irrelevant A/B/Z alphabet. The symbolic description of nucleic acid structures by the NtC classes and CANA codes can be used to analyze details of the DNA double helical arrangements (26) and now also complex RNA folds. We present structural classification of several types of RNA loops, compare the structural features of the ligand binding sites of riboswitches and the catalytic sites of ribosomes and ribozymes. These analyses are based on symbolic description of nucleic acid structures in terms of NtC and CANA codes and demonstrate the power of such a non-subjective structural analysis. Further, the analysis of dinucleotide sequences as they occur in the NtC classes representing the most common DNA and RNA architectures, A and B forms, showed that, surprisingly, they are not sequence neutral and prefer certain dinucleotide sequences.

The described classification schema of nucleic acid dinucleotides is robust, easy-to-use tool with a potential to improve the refinement and validation protocols as well as open new ways to discover and classify nucleic acid structural motifs. It opens a way for simple, intuitive graphical representation of the nucleic acid structure and as such is of general use for the experts in structural biology as well for molecular biologists and bioinformaticians.

## MATERIALS AND METHODS

### Retrieval of structures

#### Selection of DNA and RNA structures for clustering to the nucleotide conformer classes NtC

The clustering procedure was seeded by the previously defined NtC set derived from the DNA structures only. We therefore used the same selection of 2405 DNA–protein complexes and 879 structures of naked DNA used in our previous work, which was reduced to 1791 non-redundant structures containing 57 634 steps (14). For the training set of RNA dinucleotides, we selected RNA structures using a selection procedure analogical to the DNA structure selection but on a more recent PDB (27) release from 8 July 2015. We searched for crystal structures containing RNA chains at least six nucleotides long with a crystallographic resolution better than 2.5 Å, possibly containing proteins but not DNA nor DNA/RNA hybrids. We retrieved 401 RNA–protein complexes and 321 structures of naked RNA that were further treated for sequential redundancy and considered redundant if they had >90% sequence identity. Among the redundant structures, the structure with the best resolution was selected; when inconclusive, the structure with the best MolProbity score (28) was selected. To analyze similar numbers of steps in the DNA and RNA sets, we added four large ribosomal structures with the greatest sequential differences and the best available resolution (1vy5 (29), 4u4r (30), 4v88 (31), 4lnt (32)) to the RNA set. Together, we analyzed 57 634 steps from 1791 DNA structures and 57 011 steps from 327 RNA structures; PDB codes of the analyzed structures are in Supplementary Table S1.

#### Selection of structures for analysis of riboswitch and ribosome structural features

From the PDB release of 12 July 2019, we selected 104 riboswitch crystal structures with a crystallographic resolution better than 2.5 Å. At the dnatco.org server (33) the structures were then assigned their dinucleotide conformers NtC and their ligand binding sites were further analyzed. The ligand binding site is defined as nucleotides within the 7 Å radius from any riboswitch ligand non-hydrogen atom. We further analyzed the catalytic active sites of three ribosome crystal structures with the least sequence similarity and the best possible crystallographic resolution; the selected structures were from *Escherichia coli* (4ybb, (34)), *Thermus thermophilus* (4v90, (35)), and *Saccharomyces cerevisiae* (4v88, (31)). To compare crystal and cryo-electron microscopy structures, we chose three prokaryotic and three eukaryotic structures solved by the cryo-EM technique (PDB release of 2019-08-28) with the best resolution. The prokaryotic ribosomes were from *E. coli* (5afi (36) and 5mdz (37)) and from *T. thermophilus* (6gzq, (38)). The eukaryotic ribosomes were from *Saccharomyces cerevisiae* (6s47 (39) and 5mre (40)) and from *Oryctolagus cuniculus* (6r6p, (41)).

#### The fragment definition (Figure 2)

We analyzed the same near-dinucleotide fragment as previously (14) that covers geometry between torsions δ1 and δ2, i. e. between atom C5′ of the first and atom O3′ of the following nucleotide (Figure 2). However, certain inconsistencies with the classification of intercalated and non-helical structures led us to introduce three additional geometry parameters to more precisely define the sugar-sugar (C1′–C1′) and base-base (N-N) distances and the base-base mutual orientation defined as a pseudo-torsion angle between the two C1′–N glycosidic bonds (labeled μ). The geometry of the fragment is now defined by seven backbone torsions between two consecutive (deoxy)ribose rings (in Figure 2 between torsions δ1 and δ2), by two torsions at the glycosidic bonds (χ1 and χ2), and three parameters describing the mutual orientation of the two nitrogenous bases (distances NN, C’C’, and pseudo-torsion μ).

### The clustering and assignment

#### Clustering

Both old and new NtC conformational classes were newly defined using the 12 geometrical parameters as depicted in Figure 2 and described in the previous paragraph. The NtC definitions were based on hierarchical clustering using the function *hclust* of the R software processing, a beforehand calculated circularity aware distance matrix. The search for new clusters was performed on steps which were so far unassigned. The initial step of the process was generating a temporary golden set, which was constructed from potential classes containing at least eight members, and new members with the best geometry fit to the current members were added.

#### Assignment protocol

The addition of the three new geometry parameters into the fragment definition required only slight modifications of the assignment protocol relative to its previous version (14), shown schematically in Supplementary Figure S1. The measurement of Euclidean distances and calculation of votes required transformation of the C1′–C1′ and N9/1–N9/1 distances to the scale of torsional angles. The distances were scaled by a factor of 32, which was derived as the ratio between the modes of distributions of standard deviations of torsions and distances.

The assignment protocol was used to generate a self-consistent golden set using the previously derived golden set combined with the temporary golden set. The resulting golden set consisting of 6870 dinucleotide steps defined 96 NtC classes of both DNA and RNA. It was based on the analysis of 114 645 steps, ∼50% of them RNA and 50% DNA, from 2118 crystal structures. The geometry and concise annotation of the 96 NtC classes is in Supplementary Table S2A and is also available at the dnatco.org website.

#### The confal score: validation of geometric match between analyzed step and NtC classes

To assess the structural similarity between the analyzed step and a particular NtC class we have previously introduced the confal score (14). The score is calculated for each step defining geometric parameter leading to 12 values in the range from 0 (no match) to 100 (perfect match). The previous definition of confal used the arithmetic mean to calculate the value for the whole step, the current definition uses the harmonic mean for its higher sensitivity to outliers.

### Analysis of GNRA and UNCG tetraloops

To be able to assign dinucleotide conformer classes to the first and last bases of tetraloops, we identified all hexanucleotides with sequences N-GNRA-N and N-UNCG-N in our RNA set. We found 3424 such hexanucleotides and analyzed patterns of five NtC codes assigned to them. We found 1316 hexanucleotides with other than double helix forming AAxx, BBxx, ABxx or BAxx NtC classes. Some of these non-trivial repeating patterns of NtCs form tetraloops and other loops of similar size and are reported in Results.

## RESULTS AND DISCUSSION

### Overview of the NtC classes, formulation of a new structural alphabet

The newly defined ensemble of the NtC (diNucleotide Conformer) classes version 3.5 is based on the dinucleotide training set derived from both RNA and DNA crystal structures and describes local dinucleotide structures by an automated stringently geometric assignment protocol. The protocol recognizes 96 NtC classes with defined geometries and one class, NANT, for geometrically unclassified steps; all NtC classes can be browsed at the dnatco.org website. Since our previous analysis (14), 52 classes have been newly defined; they are labeled ‘N’ in Supplementary Table S2A. The new NtC classes were discovered due to two facts: (i) the analysis of both DNA and RNA structures, and (ii) a more precisely defined geometry of the dinucleotide fragment (Figure 2). The newly defined NtC classes occur mostly in non-double helical regions of RNA and to a smaller extent of DNA. As discussed below, some of these RNA-dominant NtCs can be linked to the *consensus RNA conformers* published by the RNA Ontology Consortium earlier (12). The NtC classes resulting from the earlier analysis of DNA structures (14) were all confirmed, their definitions changed insignificantly, if at all. One class, BB16, split to two, BB16 and IC06.

Of the 96+1 NtC classes, 31 occur almost solely in DNA structures (defined as <5% of RNA in a given NtC class), and 28 occur mostly in RNA structures. In 15 classes, DNA and RNA occur at comparable frequencies (measured as at least 10% of RNA or DNA dinucleotides in an NtC class). Some of these ‘amphibious’ NtC classes are quite frequent: (i) clearly the most important is the canonical A-form AA00 but some other highly populated A-like conformers, such as AA01 are also observed often. The NtC AB05 is in fact the most frequent RNA conformer with ribose in the *C2*′*-endo* pucker. Among the unusual conformers, open OP20 links two strands in DNA or RNA, typically in four-way junctions; IC01 has intercalated either a base (typically in RNA) or a drug aromatic ring in DNA. The classes found solely in DNA are all B-like conformers, which are sterically nearly impossible in RNA, also several A–B and B–A classes occur significantly more in DNA than in RNA. RNA is more prone to occur in A-like conformers and several new A-like conformational classes occur only in RNA structures, including the frequent AA08. NtC classes with the most atypical torsion combinations and geometry, which are incompatible with the double helical arrangement, are labeled OPxx. Unsurprisingly, they dominate in RNA.

Both NtC classes and CANA codes carry a limited mnemonic content but they are primarily formal identifiers. NtCs are labeled by four-letter names. When the bases are stacked, the first letter A, B or Z characterizes the combination of the sugar pucker and glycosidic torsion χ of the first nucleotide, similarly the second letter describes the second nucleotide. For the NtCs with unstacked but parallel bases, we use letters IC for *intercalated*. When the bases are unstacked, not parallel, and/or in another unusual mutual orientation we use letters OP for *open*. The last two positions of the NtC code are sequentially assigned numbers or the letter S for the first or second base in the *syn* orientation.

The assignment protocol as currently implemented relies on the standard nomenclature for the atoms defining the analyzed torsions (Figure 2), not on the residue names. Modified nucleotides can therefore be analyzed when their atom nomenclature complies with the nomenclature of the standard ribo- or deoxyribo nucleotides for atoms defining the torsions between δ and δ+1 and χ and χ+1; the list of modified residues that contain standard atom names can be found at the help pages of the dnatco.org webservice. In this context, the RNA/DNA hybrids pose no problem and are analyzed without difficulty. For instance, the structure of the nonamer (rA)5–dA–(rA)5 (5vxq, (42)) forms parallel duplexes with all adenine bases in the Hoogsteen-like base pairs and the steps classified as A-like NtC classes, hybrid hexanucleotide (dCGdCGdCG) (5ebi, (43)) forms Z form duplexes and all steps are classified as Z form NtC classes.

### Formulation of a new structural alphabet

A strictly geometric classification into the NtC classes is a powerful tool for computer-based automated structure annotation. To help a more intuitive understanding of the main structural features of the analyzed structures and to allow their graphical representation, we categorized the NtC classes into fewer groups that define the structural alphabet CANA (Conformational Alphabet of Nucleic Acids) (14).

Many newly identified NtC classes required redefinition of the original CANA codes to reflect the complexities of the RNA architectures and to unify the way how the B and A form conformers are grouped into the CANA codes. The current version 2.3 of the CANA alphabet uses 15 three-letter codes (Table 1), 14 for structurally defined and 1 for unclassified dinucleotides. The codes AAA, BBB, and ZZZ include NtCs typical for the A, B and Z forms. AAw and BBw denote NtCs with some torsion angles, mostly α and ɣ, having mutually switched typical values. AAu encompasses A-like conformers with distant but parallel bases that are partially unstacked and may be sometimes intercalated; AAu has some features common with ICL. BB2 are classes describing the BII-form. The A–B and B–A codes represent NtCs mixing features of the A and B forms, namely sugar pucker and values of the χ torsion typical for either A or B form. The code miB marks dinucleotides exhibiting some features typical for B structures, namely C2′-*endo* sugar pucker, and a high *anti* glycosidic torsion angle, but some other torsions acquire atypical values. The ICL CANA code includes NtCs with approximately parallel bases that can be intercalated by another base, drug, or amino acid residue. The code OPN contains all NtCs with bases that are not stacked, cannot be intercalated, are often distant, and point to different directions.

**Table 1.** The 14+1 CANA codes and their populations in RNA and DNA crystal structures. A complete list of NtCs with their membership to the CANA codes and the mean values of the geometry parameters, which define them (Figure 1), are in Supplementary Table S2B and on the dnatco.org website

			DNA		RNA	
The main features of the CANA codes	CANA code	# of NtC	#	**%**	#	%
A-form conformers	AAA	6	2752	4.7	32 650	57.3
A-like conformers with switched (mostly α/ɣ) torsion values	AAw	5	330	0.6	3485	6.1
A-like conformers with distant bases	AAu	3	8	0.0	816	1.4
conformers bridging A- to B-form	A-B	5	2981	5.2	1256	2.2
conformers bridging B- to A-form	B-A	8	4043	7.0	134	0.2
the most frequent B conformers, define the ‘canonical’ B form	BBB	2	22 918	39.8	8	0.0
lesser BI conformers, some with switched (mostly α/ɣ) torsion values	BBw	5	3770	6.5	4	0.0
conformers bridging BI- to BII-form	B12	2	3688	6.4	3	0.0
conformers defining the BII form	BB2	2	2866	5.0	23	0.0
minor B conformers with untypical torsion combinations	miB	6	2303	34.0	2	0.0
conformers with bases which can be intercalated	ICL	7	125	0.2	408	0.7
conformers with unstacked often distant bases	OPN	33	189	0.3	2627	4.6
conformers with one base in syn orientation	SYN	6	410	0.7	192	0.3
Z-forms	ZZZ	6	332	0.6	341	0.6
All assigned steps	--	96	46 715	81.1	41 949	73.6
Non-assigned steps	NAN	1	10 919	18.9	15 062	26.4
All steps	--	97	57 634	100.0	57 011	100.0

### NtC classes compared to the consensus RNA conformers (12)

The exact pairing between NtC and the consensus RNA conformers published by the RNA Ontology Consortium (12) is complicated by two facts: (i) differences in the fragment definitions, and (ii) different assignment protocols. While both, NtC and *suite*, cover structural information about the backbone between torsions δ1 and δ2, i.e. between atom C5′ of the first and atom O3′ of the following nucleotide (Figure 2), the suite definition lacks description of the orientation of both bases relative to the (deoxy)ribose ring and the rest of the backbone. In our description, the orientation of bases relative to the sugar rings is described by torsions χ1 and χ2 around the glycosidic bonds, the base-to-base orientation is described by three parameters: NN, C’C’ distances, and a pseudo-torsion called μ (Figure 2). Considering the above-mentioned differences in conformer definitions, we paired 38 of the 46 consensus conformers to the NtC classes.

### Annotation of selected NtC classes

#### AAA, AAw, AAu: the CANA codes of A-like dinucleotides

Newly introduced RNA structures allowed more detailed definitions of A-like conformers. Most of the new AA classes can be characterized as variations of the canonical AA00 form; they are included under the CANA code AAA. A few new AA classes have typical A-form features but some of their torsion angles, typically α and ɣ, have values switched from their typical values. These NtC classes, e.g. AA01 and AA05, are included into the CANA code AAw. All AAxx classes occur in RNA, where they build double helical segments but participate also in the single stranded RNA parts; overall, they represent almost 2/3 of all RNA dinucleotides. Most of the AAxx classes are also observed in DNA.

The frequently occurring AA08 class differs from AA00 only in two torsion angles, ϵ and β. Its likely function is to adjust helical parameters of long double helical stretches where AA08 occurs in a regularly repeating manner. AA02 is typical by its B-like high χ values. It is the only conformer with both C3′-*endo* sugar puckers occurring more in DNA than in RNA, very often in double helical regions in direct contact with TATA binding proteins; the AA02 class itself has a strong preference for the A and/or T nucleotides. In 1cdw (44), all nucleotides in a four nucleotide long double helical region acquire the AA02 conformation. Nucleotides with a preference for the T/A steps, having an A-like sugar pucker and B-like χ torsion have been noticed before and termed as TA-DNA (45). We classify structures of these nucleotides as occurring in conformation of the AA02 class. The AA05 class demonstrates the importance of the pseudo-torsion μ for discrimination of the NtC classes. Bases in AA05 are parallel but rotated in such a way that they stack less and build deformed parts of double helices or link loop to a? double helix, often between the GNRA loop and the stem (see also below).

*A–B and B–A: the CANA codes of dinucleotides bridging the A and B forms* have been described previously (14). The newly defined AB04 and frequent AB05 just represent a finer granularity of the present NtC ensemble.

#### BBB, BBw, B12, BB2, miB: the CANA codes of B-like dinucleotides

There are no important new NtC classes among the B-like NtC classes. However, we modified their assignment to the CANA codes to make them more comparable to the A-like codes. The BBB code describing the BI-form comprises now two NtCs, the canonical BB00 and a structurally similar BB01. BI-like classes with α/ɣ and other torsion value combinations switched from their most typical values were grouped under the code BBw in analogy with AAw. The codes B12 and BB2 dealing with NtC classes describing the BII form, and miB, comprising various more exotic B-like classes, are defined as before.

#### ICL: the CANA code for dinucleotides with intercalated bases

All ICL NtC classes were newly defined due to the introduction of three parameters NN, C’C’, and μ. It also led to a cleanup of some previously defined classes, the best example being the new IC06 class emerging from BB16. The bases of dinucleotides classified under the ICL code were in most cases intercalated by an aromatic ring of a drug molecule, another base, or an amino acid residue capable of van der Waals or cation-π interactions, such as aromatic residues and arginine or lysine. These conformers stabilize RNA 3D folds, they also enable the DNA i-motif by sandwiching two cytosines by another cytosine. The most abundant ICL class is IC01. It occurs mostly in RNA, often in steps with the NA sequence. It can be intercalated by another base (A558–A559 from chain B of 1j1u (46)) or by an aromatic substrate, such as by the ATP adenine intercalating between bases A16–A17 of chain C in 3ovb (47).

***OPN: the CANA code for ‘open’ dinucleotides***

comprises NtCs with variable geometries, which share a common attribute of being ‘open’, having non-stacked and often very distant bases. The OPxx NtCs are rare but they occur in regions critical for DNA and especially RNA folding and allow their formal and unbiased annotation. The geometries of the OPN conformers have unusual torsion angle combinations often far from the energetically optimal values observed in the most populated classes. OPN conformers can therefore be expected to be flexible and generally more influenced by their surroundings. This causes problems with the NtC assignment when wide distributions of the easiest-to-deform descriptors, torsions χ and three parameters measuring the mutual position of both bases, NN, C’C’, and μ, do not allow the unequivocal assignment of any NtC. The assignment protocol may need some modifications to account for the peculiar behavior of the OPN NtCs.

**Figure 3.**
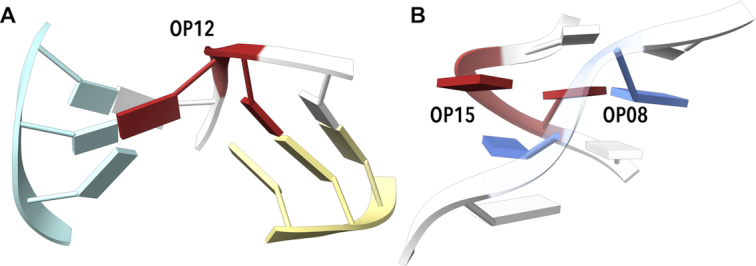
Examples of simple motifs built by open conformers. (**A**) Example of OP12 motif (red) assigned to step G110–C111 from chain B of 2pn4 (73). The step is capable of binding three sequentially distant parts of the molecule or three different chains, one in the center, one in light blue, one in light yellow. (**B**) OP15 with both bases nearly in one plane often pairs with OP08 in the opposite strand (OP15 in red, OP08 in blue, motif from sarcin/ricin domain of 28S rRNA, step G10–U11 from chain A of 1q96 (50). Drawn by ChimeraX (74).

OP03 and OP04 are frequent and structurally similar OPN classes occurring often in tetraloops. Of 221 OP03 occurrences in the RNA set, it was found 165 times in the GN step of the GNRA tetraloops; analogical numbers for OP04 are 98 GN steps of 306 occurrences. OP04 also appears in longer motifs OP04-AAxx-ICxx-OPxx or OP04-AAxx-ICxx-NANT interacting with other loops as in the case of the OP04-AA00-IC01-OP14 sequence of residues G1389-A1393 in chain 0 of structure 1vq8 (26). Another new NtC, OP11, has a slight propensity for the YR sequence. It links two distant parts of the RNA strand(s) by forming base pairs but it often bulges out the first base outside the RNA molecule to the solution to form a sharp turn of the strand. OP12 has a strong propensity for the YC or NC sequences. It forms a well-defined spatial motif with its first base (mostly Y) unpaired, interacting with sequentially distant phosphate, and stacked by the sequentially preceding base. The second base is paired and stacked between two bases, one sequentially preceding, the other distant; the stacking and pairing of the second base stabilizes close contact between two sequentially distant parts of the molecule of two RNA strands (Figure 3A). OP12 can alternatively expose the first base outside the RNA (step C20–C21 from chain D of 3vjr (48)), similarly to OP11. Various OPxx classes also form a range of motifs called platforms (17). In these platforms, the bases are parallel or nearly co-planar and interact with each other. Several conformers contain bases in this position: OP07, OP08, OP10, OP15, OP22, OP23, or most frequently OP26. OP15, observed also in DNA, prefers the GN sequence. Its bases are near-parallel and lie almost in one plane so that they can in some cases form hydrogen bonds to each other (N2 of G to O4 of U in step G23–U24 from chain G of 4ato (49)). OP15 allows a sharp turn of the backbone with short P–P distances. It often stands opposite to OP08 as in the structure of the sarcin–ricin domain from 28S rRNA (1q96 (50), Figure 3B).

#### SYN and ZZZ: the CANA codes comprising dinucleotides with one of the stacked bases in the syn orientation

Z-form NtCs are found mostly in DNA but they occur also in RNA, e.g. as in 2gxb (51). The relatively frequent and new AAS1 occurs mostly in RNA. The backbone torsions of ZZ01 are similar to those in ZZS2 but both its bases are in the *anti*-orientation. Therefore, it does not occur in the left handed double helices but as an isolated step where the RNA backbone makes a sharp turn, such as step C213–A214 from chain A of 1et4 (52) turning the chain by almost 180°. It is often found in contact with protein as in 1jbr (53). ZZ01 in the C77-G78 step from chain Y of 2gdi (54) facilitates interaction between the riboswitch and its ligand, thiamine pyrophosphate.

#### NAN: unclassified dinucleotides

The NAN CANA code contains a single NtC code NANT. It contains all dinucleotides, which were not assigned to any of the 96 NtCs with the defined geometry. There are large fractions of the unassigned steps in our data set: almost 19% of all DNA and over 26% of all RNA steps. The percentage of NANT dinucleotides is even higher when we assign dinucleotide geometries over the whole database: of nearly six million dinucleotides currently (as of 18 December 2019) in the PDB, over 30% remain unassigned as NANT class. It raises a question if such a large fraction of unclassified dinucleotides reflects (a) an inappropriate classification protocol, (b) conformationally unique steps or (c) an incompletely refined dinucleotide geometry. In the following paragraph, we aim to show that the latter option (iii) above is the most likely answer to most cases of the unassigned dinucleotides.

*(a) Does the assignment protocol miss a large portion of step geometries?* We are convinced that it does not. In our previous analysis of the DNA geometry (14), we could not assign 21% of the analyzed steps into any of the previously 44 defined NtC classes. Inclusion of the RNA-defined NtCs into the assignment process doubled the number of the defined NtC classes but the overall improvement of the assignment of DNA steps was a marginal 2%. This small improvement is in agreement with one of the conclusions from our previous analysis that stated ‘newly discovered conformer classes will be numerically small, accounting for a small fraction of the currently unassigned steps.’

*(b) Do the unassigned steps represent unique conformers?* The NtC classes can be seen as a reflection of the free energy hypersurface of nucleic acids because they are identified based on their recurrent occurrence. Therefore, it seems unlikely if not impossible that more than one fifth of the steps in the ensemble of the analyzed structures acquire high energy states with unique geometries that are not classifiable based on the known conformer classes.

*(c) Could the unassigned dinucleotides represent incompletely refined fragments of nucleic acid structures?* To look closer at the geometries of the unassigned steps we calculated how close their geometries are to geometries of the closest NtC class and how well they fit into electron density. The geometric fit was calculated as the root mean square deviation (rmsd) between the investigated dinucleotide and the geometrically closest dinucleotide from the golden set. We calculated two rmsd values, one between the torsions describing the dinucleotide fragment (Figure 2), the other between the Cartesian coordinates of the 18 atoms defining these torsional parameters. The fit to the electron densities was measured as real-space correlation coefficient (RSCC) (55) as the harmonically averaged RSCC values of the 18 atoms defining the dinucleotide geometry calculated using the phenix.real_space_correlation program (56).

**Figure 4.**
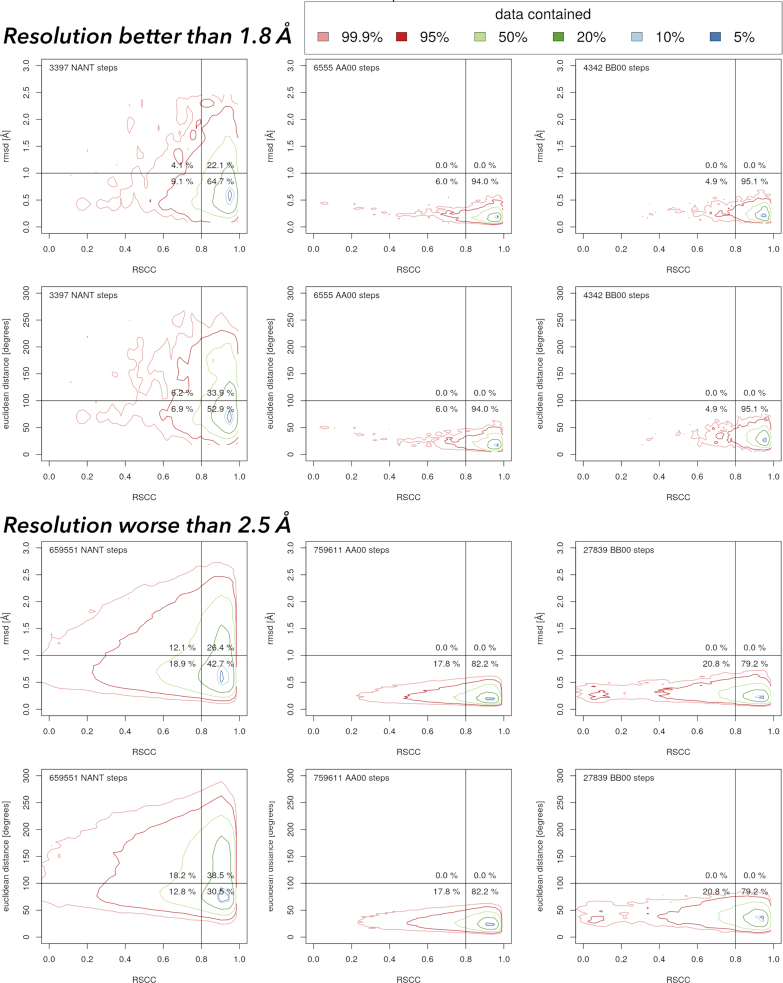
Contoured scattergrams between real-space correlation coefficient (RSCC) and two geometric measures of the fit between the dinucleotide geometry and the geometry of the closest dinucleotide in the golden set. Data were calculated for 2.6 million dinucleotides in all nucleic acid structures in the PDB with available electron density maps as of 16 December 2019. The analogical scattergrams for all NtC classes are posted at the website dnatco.org/contours. The values of the rmsd values delimiting the quadrants are somewhat arbitrary but derived from the values of the assigned dinucleotides.

The contoured scattergrams in Figure 4 show the relationships between the geometric fit to the NtC classes and the fit to electron density for dinucleotides from the PDB. The scattergrams are shown for dinucleotides assigned to the two most important NtC classes, AA00 and BB00, and also for unassigned dinucleotides. Analogical scattergrams for all 97 NtC classes can be seen at the website dnatco.org/contours. Obviously, the dinucleotides assigned to either AA00 or BB00 classes have low rmsd to their closest golden set match. Their large majority also fits well to the electron density; an RSCC correlation higher than 0.80, which is considered as a good fit, is observed for more than 90% of dinucleotides from better resolved structures and about 80% for lower resolution structures.

The scattergrams for the unclassified dinucleotides (NANT) present a more complicated picture. For the higher resolution structures, more than half of the unassigned dinucleotides fit well into the electron density (RSCC > 0.8) and at the same time have rmsd in both Cartesian and torsion spaces reasonably low; they are in the lower right quadrant. The corresponding percentage for the lower resolution structures is still >30%. By being close to a known NtC class geometrically and by fitting well into the electron density, these unassigned dinucleotides represent just incompletely refined portions of the nucleic acid structures. We believe that these nucleic acid portions can be re-refined with the help of proper refinement targets and sensibly adjusted restraints.

In contrast, dinucleotides in the upper left corner, which fit poorly to both electron density and NtC geometry, need complete refitting. However, they are rare in the higher resolution structures and represent well <20% of the unassigned dinucleotides for the lower resolution structures. These poorly fitting dinucleotides represent <5% of all dinucleotides with electron density available. The dinucleotides in the upper right quadrant, which fit well into electron density but poorly to geometries of the NtC classes as we know them now, present a challenge for the current NtC assignment algorithm. Some of these dinucleotides may represent unique conformations, others may form yet undefined NtC classes.

The scattergrams in Figure 4 confirm the clustering protocol and show that about a half of all currently unassigned dinucleotides are actually quite close to the geometries of known NtC classes and can likely be refitted if refinement protocols provide proper refinement targets for nucleic acids.

#### Sequence dependencies of NtC classes and CANA codes

Some NtC classes have a specific structural role and clearly prefer certain sequences. This is especially the case for rare classes, some of which have been discussed above; for example, BBS1 builds up almost exclusively GG steps in DNA and RNA quadruplexes. However, a fundamental question with biological consequences is whether frequent classes building up the main architectural blocks of nucleic acids are sequence-neutral or prefer certain sequences. To answer this question, we calculated the standardized Pearson residuals (SPR) (57) of the instances of 97 NtC classes in one of the 16 dinucleotide sequences, and of 15 CANA codes in one of the 16 dinucleotide sequences. We employed the protocol described in detail previously (26), the null hypothesis being that each of the NtC classes (or CANA codes) is distributed equally among the 16 dinucleotide sequences. In the following discussion, we rely only on SPR values with absolute values larger than 5 when values larger than ±3 are usually considered significantly violating the null hypothesis.

**Figure 5.**
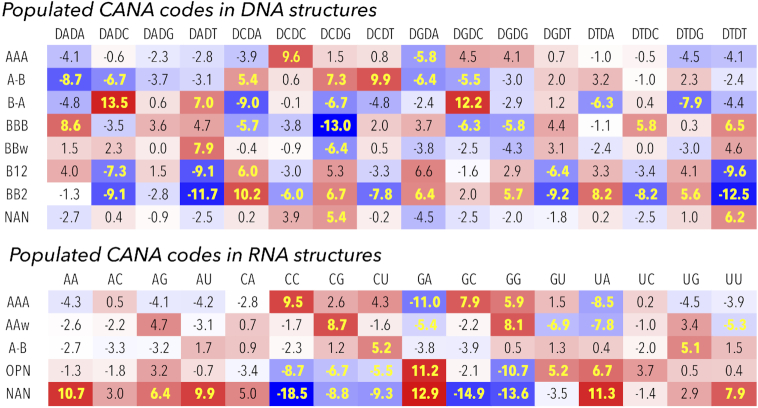
Standardized Pearson residuals (SPR) of populated CANA codes for DNA and RNA analyzed structures calculated for the sixteen dinucleotide sequences. Red (blue) color highlights overpopulated (underpopulated) instances. SPR values highlighted in yellow point to the sequence/CANA combinations where χ^2^ values are highly significant (for the 15 degrees of freedom and the significance level of 0.01 χ^2^ > 30). SPR and χ^2^ values for all CANA codes are listed in supplemental Table S3E.

Figure 5 highlights the sequence preferences for the dinucleotides classified in the highly populated CANA codes, details of the analysis are shown in detail in Supplementary Tables S3 (S3B and S3D show the statistics, S3A and S3C the numerical incidences). As we discuss further and as Figure 5 demonstrates, both structurally most important and common CANA codes, AAA in RNA and BBB in DNA, are not sequence neutral and do not occur with the same frequency in all 16 dinucleotide sequences.

#### Analysis of sequence dependencies in RNA

Virtually missing B-like conformers (CANA codes BBB, BB2 etc.) and a thin population of unstacked AAu, intercalated ICL, *syn* SYN, and Z-like ZZZ dinucleotides make the interpretation of sequence preferences easier for the RNA than DNA steps (Figure 5). The AAA code comprising the canonical AA00 class and frequent AA08 shows strong sequence preferences: overpopulation for CC, GC and GG and underpopulation for GA and UA. The deviations from the expected populations of AAA are counterbalanced by the populations of the unclassified NAN steps. The meaning of the compensation is not structural but purely statistical: AAA and NAN together represent almost 85% of all RNA data so that a sequence overpopulated in AAA is almost certainly underpopulated in NAN and *vice versa*.

Looking into incidences of the main component of the RNA architecture, AA00 class, we observe even more pronounced deviations in sequence preferences than for AAA: it is underpopulated in AA, AC, AG, AU, GA, UA, UG, UU and overpopulated in CC, CG, CU, GC, GG (Supplementary Table S3D). In other words, some sequences are more likely to be observed in the canonical A form than others, the AA00 class is not ‘sequence neutral’. To investigate the robustness of this observation, we tested whether the same sequence preferences would be observed for two subgroups of the tested RNA structures, ribosomes and no-ribosomes. Both groups revealed the same pattern of under- and overpopulated sequences with the SPR coefficients acquiring more extreme values for the ribosome structures. Sequence dependencies have recently been observed by experiments measuring the energetics of formation of various RNA duplexes (58).

For the open conformers under the OPN code, we noticed an interesting relationship between the overpopulated GA and underpopulated GG (and CC) steps. The structural meaning of these preferences is not immediately obvious but it could be related to a high propensity of G and C to form more stable double helices than A and T and therefore being more likely to form double helical than open fragments of RNA molecules.

#### Analysis of sequence dependencies in DNA

The CANA codes or NtC classes assigned to the DNA dinucleotides reveal a complicated pattern of their under- or overpopulation in the 16 sequences as measured by the SPR. The most important and in our opinion surprising are large over- and under-populations in the BI form classes BB00 and BB01 (CANA BBB). These two NtCs are preferred in A/T rich AA, TT, and TC steps and strongly underrepresented in CG but also in CA, GC, GG. The prevailing B-DNA conformation, BI, is therefore not neutral to dinucleotide sequences as was the case for the A form in RNA.

The BII form described by CANA code BB2 disfavors the NY steps and prefers NR ones including CA and TA. The preference for the TA sequence is important in the light of two previous reports about the sequence and structure behavior of the histone-bound DNA: the TA periodicity statistically inferred from the genomic data (59) and the ten step periodicity of the BB2 code in crystals of nucleosome core particles (26). The CANA code B12 bridging BI and BII conformations shows behavior virtually identical to those of BB2.

Some less populated B form-related NtC classes show sequence preferences (Supplementary Tables S3). For instance, in more than half of all cases, BB15 has the NC sequence. In more than two thirds of all instances, BB16 starts with pyrimidine. BB12 belonging to the miB code has a complementary propensity, often ends with pyrimidine and starts with purine: the NY sequence was observed 320 times, RY 239 times out of 456 instances. Certain complementarity in sequence propensities between BB12 and BB16 expresses itself as a quite frequent trinucleotide motif BB12–BB16 with sequences ACA, ACT, or in general ACN. These observations are however just statistical propensities and an inverted motif, BB16–BB12, was observed as well. Both motifs accommodate deformations of the double helix induced by interactions with proteins.

In contrast to the SPR indicated sequence preferences of unassigned steps in RNA, which mirror the A form preferences, the SPR values in DNA show only mild sequence dependencies with the exception of overpopulated CG and TT. This means that geometries of incompletely or incorrectly refined steps are not a function of the sequence in DNA structures.

### Structural analysis using NtC classes and CANA codes

#### Structural analysis of tetraloop motifs

**Figure 6.**
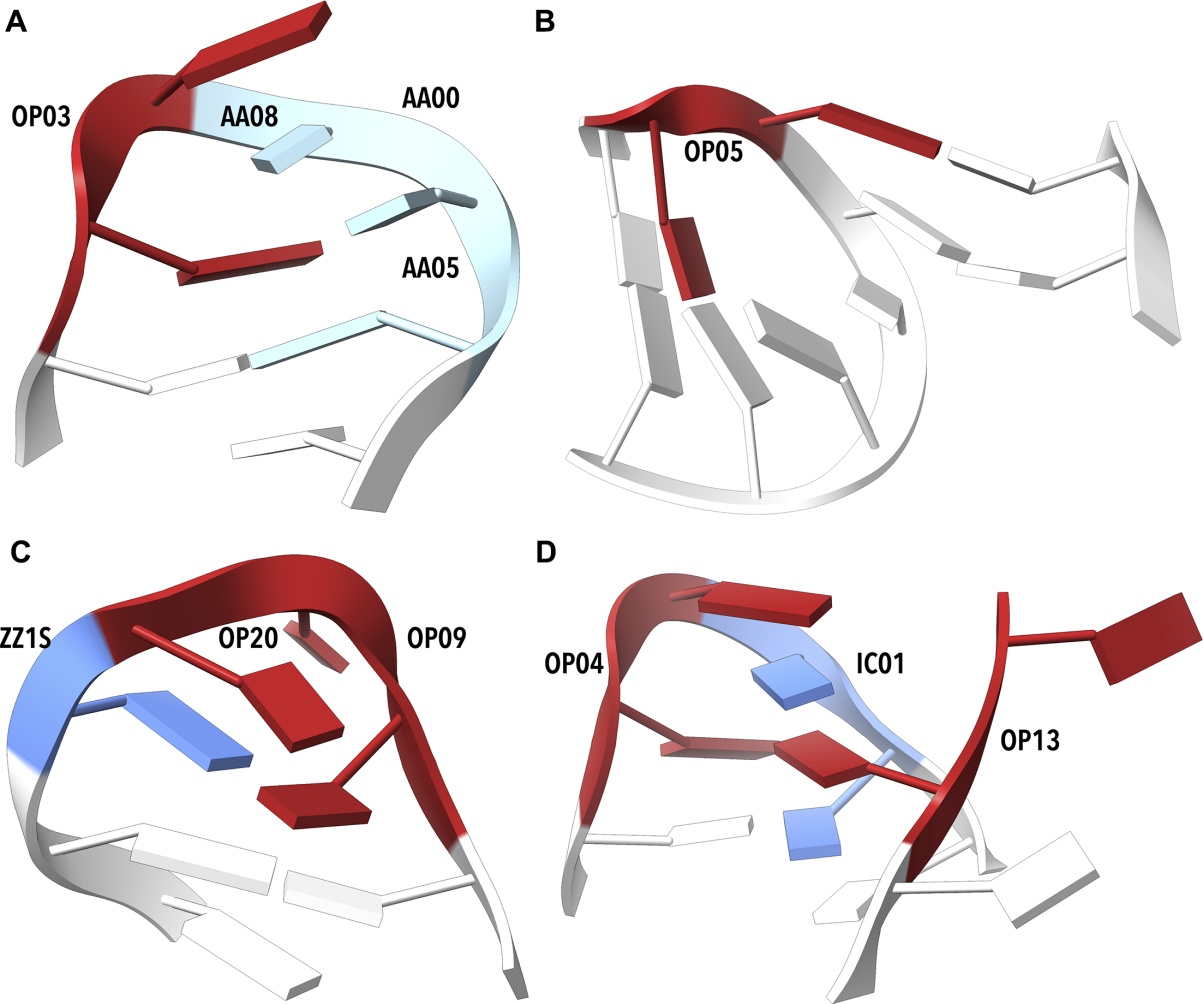
Examples of tetraloop and tetraloop involving motifs. (**A**) Tetraloop from 4lvz (75) contains OP03 (step G59-A60), followed by a series of A-like NtC classes. (**B**) The open conformation OP05 preceding the actual tetraloop G2738–A2739–G2740–A2741 in the step C2737–G2738 of 1vq8 (76) enables a kissing loop motif to a distant part of the molecule. (**C**) Two OPN (OP09 and OP20), are adjacent to a ZZ1S step in nucleotides U2144–C2145–C2146–G2147 of (77). (**D**) The loop from 4qvi (77) built by OP04 (step G2168–A2169) and IC01 (step A2170–A2171) pairs with distant base (A2119), a part of OP13. Drawn by ChimeraX (74).

We searched the RNA structures for two of the most common tetraloop sequences, GNRA and UNCG. The GNRA sequence pattern was found 392 times in our set of RNA structures. It is described by three steps: the first one, GN, is frequently formed by the OP03 or OP04 class (171 and 103 cases, respectively) and is followed by dinucleotides in AAxx classes. The three NtCs assigned to the GNRA sequence are often *followed* by the rare A-like AA05, occasionally AA08 or it is unassigned so that the motif GNRA-N can be summarized by the NtC sequence OP03/OP04-AAxx-AAxx-AA05, which was observed in 84 cases (Figure 6A). It is likely much more prevalent as we also observe OP03/OP04-X-X-NANT motifs (149×) with geometries of many unassigned steps close to AA05 or AA08. The AA05 conformer is related quite closely to the tetraloop structures as it occurs 120 times at the end of the GNRA sequence.

A less frequently occurring but structurally distinct GNRA tetraloop motif has been observed before (60). It contains OP05 *preceding* the GNRA tetraloop sequence. It is usually followed by AAxx classes with bases stacked to the last bases of the motif. This N-GNRA motif can be generalized as OP05-AAxx-AAxx-AAxx (Figure 6B). We also identified a few GNRA tetraloops containing a step assigned to ZZ1S as proposed earlier based on NMR models (60), a crystal structure example can be found in 4lgt (61).

The UNCG tetraloop containing a Z-like conformer at the CG position has been named Z-turn (62). The whole tetraloop has a quite complicated geometry described by the NtC sequence OP09-OP20-ZZ1S (Figure 6C). The motif can be followed by AAS1 or another conformer with the first base in the *syn* orientation. There are other rarely occurring UNCG motifs such as the twice observed OP03-AAxx-AAxx-AAxx or AAxx-OP03/OP04-AAxx-ICxx (Figure 6D).

The search for recurrent NtC sequences forming GNRA and UNCG tetraloops led to the identification of other types of loops like UCGC, GUCC, or UCGR. They all contain an OPN conformer forming a turn necessary to close the loop. For instance, clusters named C02 and C05 and classified as different in (60) share the central open conformer ZZ1S and their structures differ in the stem parts; the corresponding NtC motif for the loop itself is OP09-OP20-ZZ1S. Similarly, clusters named C01, C03, C06, and C09 in (60) share the turning conformer OP03 or OP04 followed by AAxx forming a motif OP03/OP04-AAxx-AAxx-AAxx.

The searches for recurrent sequences of the NtC classes exemplified above demonstrate a potential to discover new motifs or their structurally more detailed description. A motif originally identified by a base pairing or base interaction pattern can match its NtC-based mate but often the finer granularity of an NtC-based classification leads to a split into several structurally distinct motifs. On the other hand, an advantage of motif searches based on base pairing is in their ability to identify motifs regardless of gaps in the nucleotide sequence. A subsequent assignment of the NtC symbols to the motif dinucleotides is easy and can provide their more detailed structural characterization.

#### The structural annotation of the binding sites of riboswitches

In 104 selected riboswitch structures, we analyzed 9,045 steps and 1,777 of them were found in the binding sites defined as nucleotides within 7 Å off any of the ligand non-hydrogen atoms. The riboswitches contain a high variability of ligands of diverse chemistry and size from metal cations to large coenzymes or cofactors. The NtC assignment for the riboswitch structures is summarized in Table 2 and in more detail in Supplementary Table S4A. The distribution of the NtC classes found in these structures differs from the NtC distribution in all analyzed RNA steps by a lower fraction of unassigned steps; NtC NANT: 20% in riboswitches, 26% in all RNA steps. The difference can be explained by a higher average resolution of the riboswitch structures compared to the resolution of all RNA structures. Except for this difference, the overall distribution of frequencies of the NtC classes in riboswitch and all RNA steps is similar.

**Table 2.** The incidences of the NtC classes most frequently occurring in the riboswitch crystal structures inside and outside of the binding sites. The values of the standardized Pearson residuals (SPR) measure the size of the populations of the individual NtCs in the binding sites relative to the rest of the molecules; positive values indicate over-population of an NtC inside the active site

NtC	Inside	Outside	SPR
AA00	766	3909	−8.1
AA01	29	290	−4.8
AA05	21	32	3.7
AA12	23	30	4.4
AB04	19	14	5.5
IC01	14	11	4.6
OP05	22	5	8.1
OP15	33	10	9.4
NANT	460	1374	6.6
Rest of NtCs	390	1593	--
Sum	1777	7268	--

**Figure 7.**
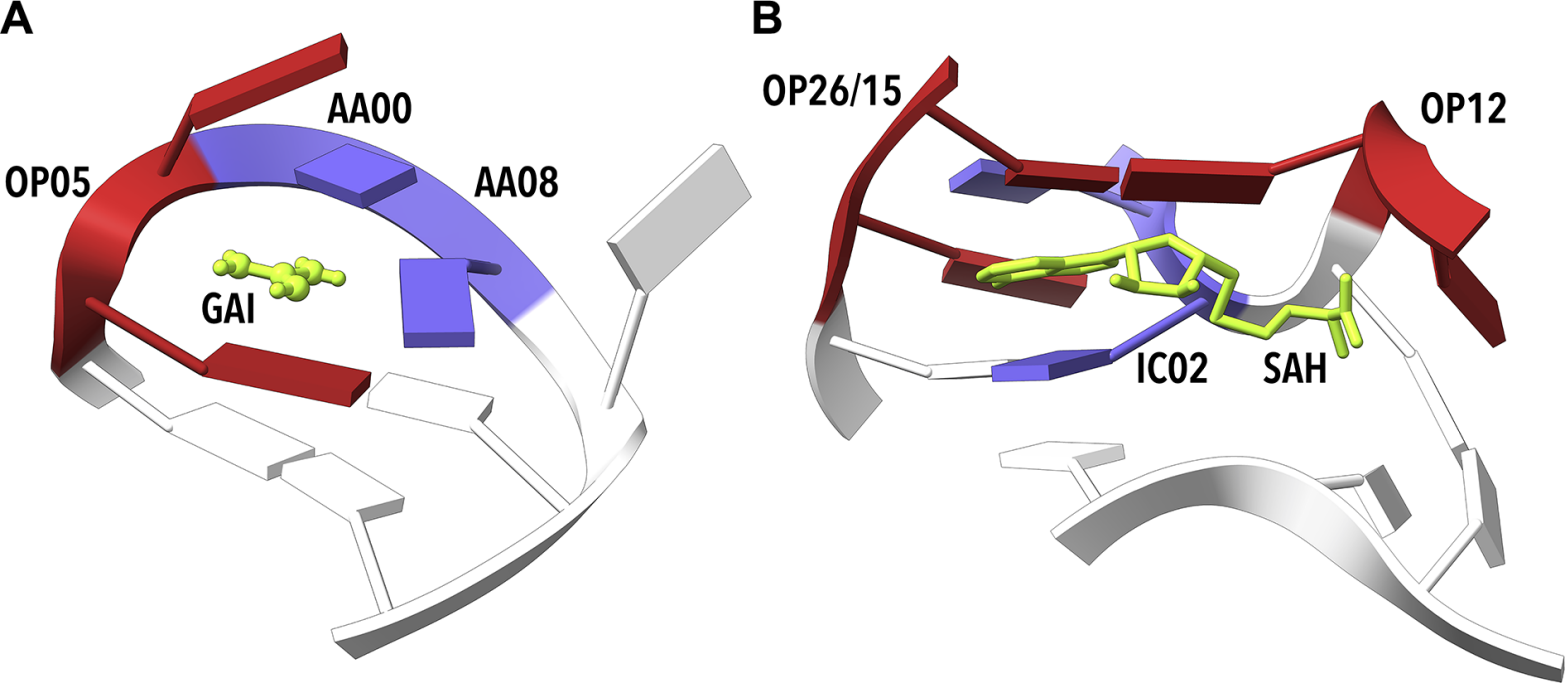
Examples of riboswitch binding sites. (**A**) Guanidine II riboswitch bound to guanidine (GAI, 5ndh (63)). Step G6–A7, which facilitates GAI (green) binding, was assigned to OP05. (**B**) *S*-Adenosyl homocysteine (SAH) riboswitch (3npq, (64)) binding SAH. Step G15–C16 in the close proximity of an adenosyl group in the ligand SAH (green) is unassigned but very close to OP26 and OP15. Step C28–A29 was assigned to IC02 (blue) and step G31–C32 was assigned to OP12. The structure of these NtCs allows binding of a large ligand, in this example SAH, via intercalation and stacking. Drawn by ChimeraX (74).

We tested whether some NtC classes occur more frequently in the binding sites or in the rest of the riboswitch molecules. To compare the numerical incidences of individual NtCs inside and outside the binding sites, we calculated values of SPR. As in the case of our sequence analysis, high absolute values of SPR indicate violation of the equal distribution inside and outside the active site; here positive (negative) SPR values indicate an over-population (under-population) of a given NtC in the active site relative to the rest of the molecule. Table 2 shows that rare dinucleotide conformations AB04, IC04, OP05, OP15 and NANT are over-populated in the riboswitch active sites (SPR analysis for all NtCs is in Supplementary Table S4A). In contrast, two common A-form NtCs, AA00 and AA01, are highly under-populated in the binding sites relative to the rest of the molecules. A high occurrence of rare and mostly non-helical conformers and fewer A-like ones in the binding sites is required to build complicated molecular architectures to achieve specific binding of ligands. Figure 7 depicts binding sites of two riboswitches, guanidine II (5ndh, (63)) and *S*-adenosylhomocysteine (3npq, (64)) riboswitches.

#### The structural annotation of the catalytic sites of selected ribozymes and ribosomes

Gaines *et al.* grouped several ribozyme types under the L-platform or L-scaffold (65) that should represent a common framework for five native RNA ribozymes and one artificial DNAzyme. Assignment of the NtC classes to the active sites of some of these L-platform ribozymes (4oji (66) 2oue (67) 5v3i (68) 2oue (69) 5k7c (70)) and one DNAzyme (5xm8 (71)) is shown in Supplementary Table S5. The assignment does not show any obvious pattern of preferred NtC classes or CANA symbols except that they seem to copy the preferences observed in the overall sample of RNA (preference for AAxx) or DNA (preference for BBxx) dinucleotides, and a more detailed analysis is clearly needed. A computational approach to analysis of the nucleotides around active site has been published recently (72) confirming their intrinsically dynamic behavior.

#### The ribosome active sites

We analyzed three ribosome structures, 4ybb (34), 4v90 (35) and 4v88 (31) that contained 23 992 steps (Table 3, Supplementary Table S4B). Of these, 112 steps were found to be within the 10 Å distance from the catalytically active adenine nucleotide (residue 2451 in the large subunit of prokaryotic ribosomes and 2820 in the eukaryotic one). As in all RNA structures, prevailing conformers in the ribosome structures are AA00 and AA08 (30 and 11 occurrences near the active sites, respectively). Open and other scarcely occurring NtC classes are rare in the whole structures as well as in the active sites; most of these classes are not present in the active site at all. However, a closer look at the geometries of the unassigned dinucleotides in the active site indicates that these rare classes can be much more prevalent there: out of 51 cases of the NANT class, 37 dinucleotides are within 1 Å root mean square deviation from one of the open or intercalated NtC classes; additional 10 could potentially be assigned to the A-like classes. It indicates incomplete refinement of residues critical for the ribosome catalytic activity.

**Table 3.** The incidences of the NtC classes with significant standardized Pearson residuals (SPR) values found in the selected ribosome crystal and cryo-electron microscopy structures. Positive SPR values indicate overpopulation of an NtC in the X-ray structures

NtC	X-ray	Cryo-EM	SPR
AA00	8415	9768	3.1
AA03	299	96	12.4
AA04	996	732	10.4
AA08	3109	4586	−9.4
AA06	176	331	−4.8
AB05	425	403	3.5
OP03	66	154	−4.6
OP04	147	96	4.8
NANT	6933	8926	−4.9
Rest of NtCs	3426	3829	--
Sum	23 992	28 921	--

We compared frequencies of the NtC classes in the three ribosome crystal structures and selected cryo-EM structures to look at possible differences in the refinement strategies of crystal and cryo-EM data. Table 3 shows that some highly populated NtC classes, such as AA04, do show significantly different frequencies between the compared structures. Overpopulation of the unassigned conformers NANT in cryo-EM structures suggests that refinement of these structures of generally lower resolution is more challenging. Incidences and SPR values for all NtC classes are in Supplementary Table S4B.

## CONCLUSIONS

By analyzing sequentially nonredundant RNA and DNA crystal structures, we formulated a set of 96+1 dinucleotide conformer classes, NtC. The 96 NtCs describe the local geometry of both RNA and DNA, the last class is reserved for the geometrically unassigned dinucleotides. The automated procedure assigning the NtC classes is a substantial update of the previously published algorithm (14). The geometry of the analyzed dinucleotide fragment is now described more robustly (Figure 2), which allows to differentiate the conformations of analyzed dinucleotides more precisely.

About 30% of almost six million steps found currently (December 2019) in the whole PDB cannot be assigned to any NtC class (Table 1, Supplementary Table S2A). However, as we show in scattergrams of Figure 4, up to a half of the unclassified dinucleotides (NtC class NANT, CANA code NAN) fit well into the electron density (RSCC > 0.8) and at the same time have geometries reasonably close to the geometry of the known NtC classes (rmsd < 1 Å). We suggest that these unassigned dinucleotides represent just incompletely refined portions of the nucleic acid structures that can be re-refined with help of proper refinement targets and sensibly adjusted restraints. Only less than 20% of the unassigned dinucleotides, which fit poorly to electron density and are distant from any known NtC geometry, probably need complete refitting. However, these dinucleotides with most likely incorrectly determined geometries still represent almost 5% of all dinucleotides.

The 96+1 NtC classes are grouped into 14+1 codes of the structural alphabet CANA (Conformational Alphabet of Nucleic Acids, Table 1) that enables symbolic annotation of the prominent structural features of nucleic acids. The search for occurrences of nontrivial sequences of the NtC classes and/or CANA codes in the RNA-containing structures resulted in the identification of several types of RNA tetraloops and loops of similar size (Figure 6), some of them unobserved before. An analysis of the active sites of riboswitches (Table 2, Figure 7) and of the catalytic sites of ribosomes (Table 3) characterized their structural features in symbols that are easy to investigate further.

The number of analyzed structures and steps (57 634 steps from 1791 DNA structures and 57 011 steps from 327 RNA structures) allowed for a statistical analysis of the sequence preferences of highly populated NtC classes and CANA codes. Calculation of the standardized Pearson residuals (SPR) brought surprising and statistically significant results (Figure 5). Quite remarkably, the AAA CANA code as well as its, by far the most populous, NtC class, AA00, which forms the scaffold of RNA structures, are not sequence neutral, (supplement Table S3D). AA00 is overpopulated in certain sequences (CC, CG, CU, GC, GG), and underpopulated in others (AA, AG, AU, GA, UA, UU). DNA molecules with more uniform, mostly double helical architecture show more complex NtC/sequence dependencies than RNA with complex 3D folds. The DNA most frequent NtC class, BB00 is, similarly to AA00 in RNA, not sequentially neutral but it prefers AA, GA, AG, and disfavors GC, AC, and mostly CG sequences. The CANA code BB2 describing the BII form shows the strongest sequence preferences: it favors CA, CG, GA, GG, TA, TG, and disfavors AC, AT, CC, CT, GT, TC, TT. The many sharp sequence preferences of steps in the BII form are quite remarkable in the light of the importance of this form for bending DNA in the nucleosomes (26,58).

The reported automated assignment of NtC classes and CANA codes to dinucleotides in any RNA or DNA structure, which is available with many other functionalities at the dnatco.org web service, provides a powerful tool for an unbiased annotation and validation of nucleic acids with a potential to improve their refinement. It opens new ways to discover and classify nucleic acid structural motifs and enables simple, intuitive graphical representation of the nucleic acid structure and we believe it is of general use for the experts in structural biology as well for molecular biologists and bioinformaticians.

## Supplementary Material

gkaa383_Supplemental_File
